# Efficacy and inflammatory levels (IL-6, IL-10) of adjuvant application of vitamin-A in the treatment of pediatric *Mycoplasma pneumoniae* pneumonia: a systematic review and meta-analysis

**DOI:** 10.3389/fped.2024.1345458

**Published:** 2024-05-27

**Authors:** Limei Cao, Mali Lin, Fang Sheng, Buqing Chen, Chuanze Hu

**Affiliations:** Pediatric Ward 1, Jinhua Maternal & Child Health Care Hospital, Jinhua, China

**Keywords:** vitamin A, pediatric *mycoplasma pneumoniae* pneumonia, inflammation level, meta-analysis, infection

## Abstract

**Objective:**

The purpose of this study is to evaluate the efficacy of Vitamin A (VitA) as an adjuvant therapy for pediatric *Mycoplasma Pneumoniae* Pneumonia (MPP) through meta-analysis, and to investigate its impact on inflammation levels (IL-6, IL-10), in order to explore the role of VitA in pediatric MPP.

**Methods:**

Using a systematic literature search method, relevant research literature is searched, and RCT studies that meet the requirements are selected based on preset inclusion and exclusion criteria. Then, a quality evaluation was conducted on the included literature, and meta-analysis was used to calculate the combined effect values of mortality rate, hospital stay, lung rale disappearance time, cough duration, fever duration, IL-6 and IL-10 levels, and heterogeneity analysis was conducted. The levels of IL-6 and IL-10 represent the inflammatory levels in pediatric MPP patients, and exploring their changes has significant implications for the anti-inflammatory effect of treatment.

**Results:**

A total of 10 RCT studies were included, with a total sample size of 1,485, including 750 cases in the control group and 735 cases in the observation group. The meta-analysis results of this study showed that there was a significant difference in the total clinical efficacy of using VitA adjuvant therapy compared to the control group without VitA [OR = 3.07, 95%CI = (2.81, 4.27)], *P *< 0.05. However, there was no significant difference in the adverse reaction rate between the use of VitA as an adjuvant therapy and the control without VitA [OR = 1.17, 95%CI = (0.61, 2.27)], *P *> 0.05. At the same time, the hospitalization time [MSD = −0.86, 95% CI = (−1.61, −0.21)], lung rale disappearance time [MSD = −0.78, 95%CI = (−1.19,−0.51)], cough duration [MSD = −1.07, 95%CI = (−1.41, −0.71)], and fever duration [MSD = −0.47, 95%CI = (−0.72, −0.23)] using VitA as an adjuvant treatment were obviously lower. In addition, the meta-analysis outcomes also showed that the use of VitA adjuvant therapy can significantly reduce IL-6 [MSD = −1.07, 95%CI = (−1.81, −0.27)] and IL-10 [MSD = −0.13, 95%CI = (−0.31, 0.12)] levels. This indicates that the application of VitA in pediatric MPP also has the effect of reducing inflammatory response.

**Conclusion:**

Based on the meta-analysis results, VitA adjuvant therapy can significantly improve the clinical symptoms of pediatric MPP patients, shorten hospitalization time, promote the disappearance of lung rales, and alleviate cough and fever symptoms. In addition, VitA adjuvant therapy can effectively reduce inflammation levels, indicating its potential role in inhibiting inflammatory responses. In clinical practice, VitA adjuvant therapy for pediatric MPP can be promoted as a potential treatment option.

## 1 Introduction

Pediatric pneumonia is one of the common respiratory infections in children, and its incidence rate remains high worldwide. Although the treatment methods for pediatric pneumonia are constantly improving, they still face a series of challenges, including reducing mortality and improving treatment effectiveness (1). In recent years, the auxiliary application of VitA as a new strategy for the treatment of *Mycoplasma Pneumoniae* Pneumonia (MPP) has attracted attention from the academic community and clinical doctors. VitA, as a lipid soluble vitamin, plays an important role in maintaining normal physiological functions, enhancing immunity, and cell differentiation. Early studies have found that VitA not only improves children's vision and growth, but also enhances their immune response to pathogenic microorganisms, which may help treat respiratory infections (2). However, there is currently a lack of consistent conclusions regarding the efficacy and mechanism of VitA as an adjuvant therapy for pediatric MPP.

Meta analysis, as a statistical method, can systematically synthesize and analyze the results of multiple independent studies to obtain more objective and comprehensive conclusions. Therefore, this study aims to comprehensively evaluate the efficacy of adjuvant use of VitA in the treatment of pediatric MPP through meta-analysis, and further explore the regulatory effect of VitA on inflammation levels, aiming to provide more reliable evidence for clinical treatment. This paper will include published research literature on the adjuvant treatment of pediatric MPP with VitA, and conduct a systematic evaluation and meta-analysis of the clinical total response rate, adverse reactions, length of hospital stay, time to disappearance of lung rales, duration of cough, duration of fever, and inflammatory levels (IL-6 and IL-10). It hopes to provide more comprehensive and scientific guidance for the treatment of pediatric MPP through this study, promote the rational application of VitA in pediatric respiratory infections, and provide reference for further related research.

## 2 Materials and methods

### 2.1 Literature search

Based on the research topic, determine the keywords and terminology that cover the research content, including “pediatric MPP”, “VitA”, “adjuvant therapy”, “Meta analysis”, “IL-6”, “IL-10”, etc. Select literature database platforms such as PubMed/MEDLINE, Embase, Web of Science, and CNKI for literature retrieval. When searching, construct a reasonable search strategy based on the determined search keywords and terms. For example: (“pediatric MPP” or “pediatric pneumonia”), (“VitA” or “Vitamin A”), (“Adjuvant therapy” or “Adjuvant application”), (“Meta analysis” or “System evaluation”), (“IL-6” or “Interleukin-6”), and (“IL-10” or “Interleukin-10”). In addition, besides of database retrieval, relevant literature that has not been retrieved is also searched for through manual searches of references, journal catalogs, and conference papers.

### 2.2 Criteria for inclusion and exclusion of literature

Inclusion criteria: ① The study subjects were children with Mycoplasma pneumonia (aged between 1 month and 14 years old); ② The observation group was treated with VitA alone. ③ Clinical trials, cohort studies, case-control studies, or randomized controlled trials that did not use themselves as controls; ④ The literature must include data related to clinical total response rate, adverse reactions, hospital stay, lung rale disappearance time, cough duration, fever duration, IL-6 and IL-10 levels, and be accessible.

Exclusion criteria: ① Patients in the intervention group received additional medication on a routine basis; ② Children with congenital genetic diseases or defects, such as children with diabetes, tuberculosis, Kawasaki disease, kidney disease and other serious diseases were excluded; ③ Premature infants and patients with chronic lung disease were excluded; ④ Animal experiments, literature review, etc.; ⑤ Although a randomized control was conducted, the study used itself as a control; ⑥ There were no clear indicators of treatment effectiveness.

### 2.3 Data extraction

Data extraction is mainly completed independently by two researchers with rich work experience. Before starting literature data extraction, a data extraction table needs to be developed first. The table should include various data and information that needs to be extracted, such as study design, sample size, intervention measures, control group situation, primary outcome indicators, secondary outcome indicators, outcome data, etc. Developing tables in advance can ensure the orderliness and comprehensiveness of data extraction. According to the pre established data extraction table, relevant data and information are extracted from the included literature. After extraction, the quality of the extracted data is evaluated to check its accuracy and completeness. If necessary, contact the author of the original study to obtain further data information.

It is worth noting that the age range of pediatric MPP is between 1 and 14 years old, with a large age range. The differences in therapeutic effects between different age groups should be analyzed. However, most of the included literature did not mention the specific age distribution, making it impossible to conduct further subgroup analysis.

### 2.4 Statistic analysis

Literature quality: In the Cochrane risk bias assessment, there are seven bias indicators including random sequence generation and random allocation concealment. Each bias item is classified as “low risk bias”, “high risk bias”, or “unclear”. Based on the evaluation results, the credibility of the included study can be graded, and the higher the score, the better the quality.

Selection of statistical indicators: Relevant outcome indicators were extracted and included in the literature, mainly including clinical total response rate, adverse reactions, hospital stay, lung rale disappearance time, cough duration, fever duration, IL-6 and IL-10 levels, etc. Select $\bar{x}\pm s$ or *n* (%) based on the characteristics of the data type.

Heterogeneity analysis: Q-test is used to test whether there are significant differences between different research results. If the *p*-value of the Q-test is less than the pre-set significance level (usually 0.05), heterogeneity is considered to exist. *I*^2^ is an indicator that measures the degree of heterogeneity and represents the percentage of variation between different research results. The range of *I*^2^ values is 0%–100%, with larger values indicating higher heterogeneity. Usually, an *I*^2^ value below 25% is considered low heterogeneity, 25%–50% is moderate heterogeneity, 50%–75% is high heterogeneity, and over 75% is extremely high heterogeneity. The weighted mean difference (WMD) and 95% CI are used as the combined statistics.

Sensitivity analysis: Use sensitivity analysis to evaluate the robustness and reliability of research results. Specifically, the consistency between the fixed effects model and the random effects model was determined by comparing the indicators of the observation group (O) and the control group (C). The more similar the results obtained from fixed and random effects, the higher the robustness and reliability of the study.

## 3 Results

### 3.1 Literature inclusion results

**Figure 1:**
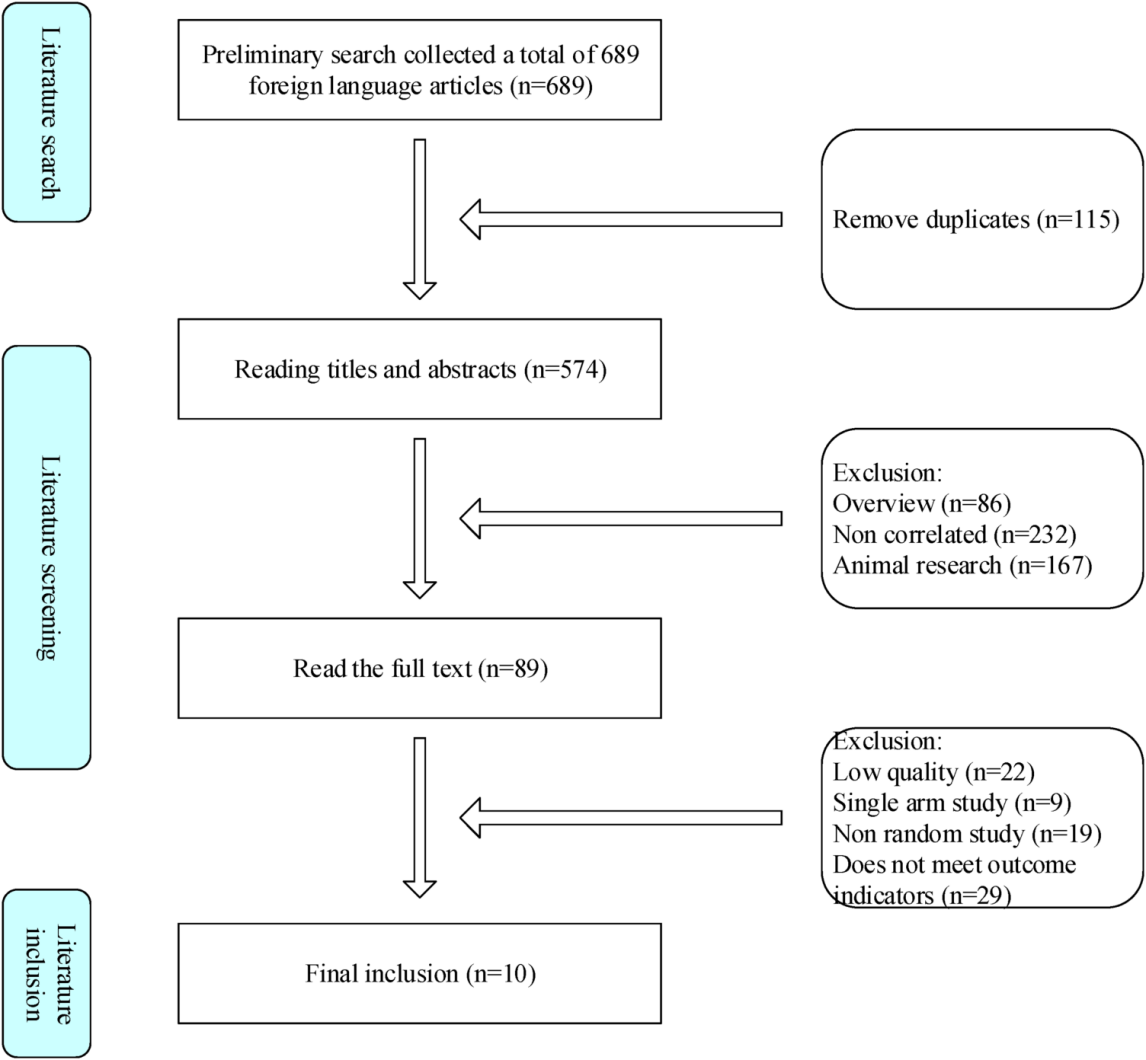
Literature inclusion process.

This study followed a search strategy and collected 689 literature from various data platforms, with 576 remaining after removing duplicates. After preliminary screening of the literature by reading the title and abstract, and fine screening of the entire text, it was ultimately included in 10 related studies (Figure 1).

### 3.2 General characteristics of literature

This study includes 10 research articles, all of which use a randomized controlled trial (RCT) study design. A total of 750 cases of C and 735 cases of O were included, resulting in a total sample size of 1,485 cases included in the study. In these included literature, O received different doses and administration methods of VitA treatment, including different doses ranging from 4,500 international units (IU) per week to 25,000 IU per week, as well as weekly and daily oral administration. The outcome indicators cover clinical total response rate, adverse reactions, hospital stay, time for disappearance of lung rales, duration of cough, duration of fever, and inflammatory levels (IL-6, IL-10). Table 1 shows the specific situation.

**Table 1:** General characteristics of included literature.

Literature	Research design	C's cases	O's cases	O's VitA treatment plan	Outcome indicators
Qin et al. (3)	RCT	46	46	4,500 IU × 7 days	1, 7
Hu Zhihong (4)	RCT	150	150	5,000–15,000 IU × 7 days	1–6
Qianqian (5)	RCT	50	50	10,000 IU × 7 days	1, 7
Li et al. (6)	RCT	128	128	4,500IU × 7 days	1, 3–6
Fengji (7)	RCT	60	40	25,000 IU × 2 days	1, 7
Huanli (8)	RCT	43	43	4,500 IU, po, qd	1, 2, 7
Zengfang and Zhaopong (9)	RCT	60	60	4,500 IU × 7 days	4, 5
Chengli (10)	RCT	23	23	4,500 IU × 7 days	1, 4, 5
Lisal et al. (11)	RCT	48	50	50,000 IU: <1 year old; 1,00,000 IU: >1 year old	2, 4, 7
Rodriguez et al. (12)	RCT	142	145	50,000 IU: <1 year old; 1,00,000 IU: >1 year old	2, 4, 6

Among the outcome indicators, 1 clinical total response rate, 2 adverse reactions, 3 hospitalization time, 4 lung rale disappearance time, 5 cough duration, 6 fever duration, and 7 inflammation levels (IL-6, IL-10).

**Figure 2:**
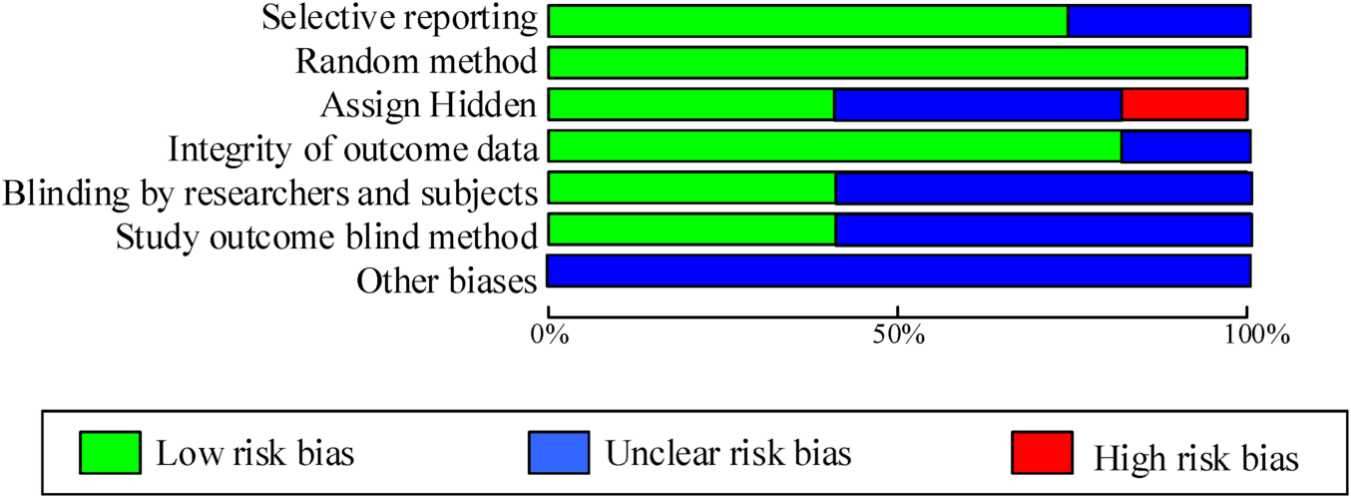
Literature quality evaluation.

As shown in Table 2; Figure 2, 7 articles have reported methods for generating random sequences; 4 articles have implemented allocation concealment; 4 articles have implemented blind methods for researchers and subjects; 4 articles achieved blind evaluation of research outcomes; 8 articles reported on loss of follow-up and follow-up, and all included articles did not report any other biases. Overall, most of the included studies have taken corresponding measures in terms of randomization, blinding implementation, and completeness of outcome data, resulting in high research quality and reliability.

**Table 2:** Bias risk assessment for inclusion in the study.

Literature	Selective reporting	Randomization method	Allocation concealment	Integrity of outcome data	Blinding by researchers and subjects	Study outcome blind method	Other biases
Qin et al. (3)	?	+	+	+	?	?	?
Hu Zhihong (4)	+	+	?	+	?	?	?
Qianqian (5)	+	+	+	+	+	+	?
Li et al. (6)	?	+	−	?	?	?	?
Fengji (7)	+	+	+	+	+	+	?
Huanli (8)	+	+	+	+	+	+	?
Zengfang and Zhaopong (9)	+	+	?	+	+	+	?
Chengli (10)	+	+	−	?	?	?	?
Lisal et al. (11)	?	+	?	+	?	?	?
Rodriguez et al. (12)	+	+	?	+	?	?	?

“+” indicates “low risk bias”; “−” indicates “high risk bias”; “?” indicates “unknown risk bias”.

### 3.3 Meta analysis results

#### 3.3.1 Clinical total effective rate

**Figure 3:**
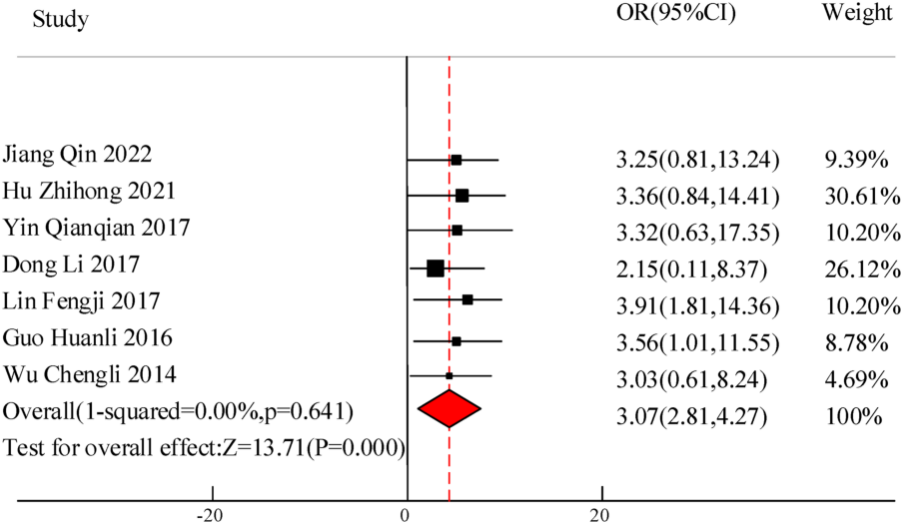
Clinical total effective rate forest diagram.

A total of 7 studies were included in this study to compare the overall clinical efficacy of VitA as an adjuvant therapy for pediatric pneumonia. Although all 7 studies evaluated “clinical efficacy”, there are certain differences in the specific efficacy evaluation. The overall evaluation method was clinical treatment effectiveness/total number of people * 100%. In this study, to eliminate the heterogeneity caused by the efficacy evaluation criteria, weighted calculation was used to obtain the overall efficacy. The results showed homogeneity in the mortality rate between the C and experimental groups (*I*^2 ^= 0%, *P *= 0.641), so a fixed effects model was used for comprehensive evaluation. The conclusion shows that there is a significant difference in the overall clinical efficacy of using VitA as an adjuvant therapy compared to C without VitA [OR = 3.07, 95% CI = (2.81, 4.27)], *P *< 0.05 (Figure 3).

#### 3.3.2 Adverse reactions

**Figure 4:**
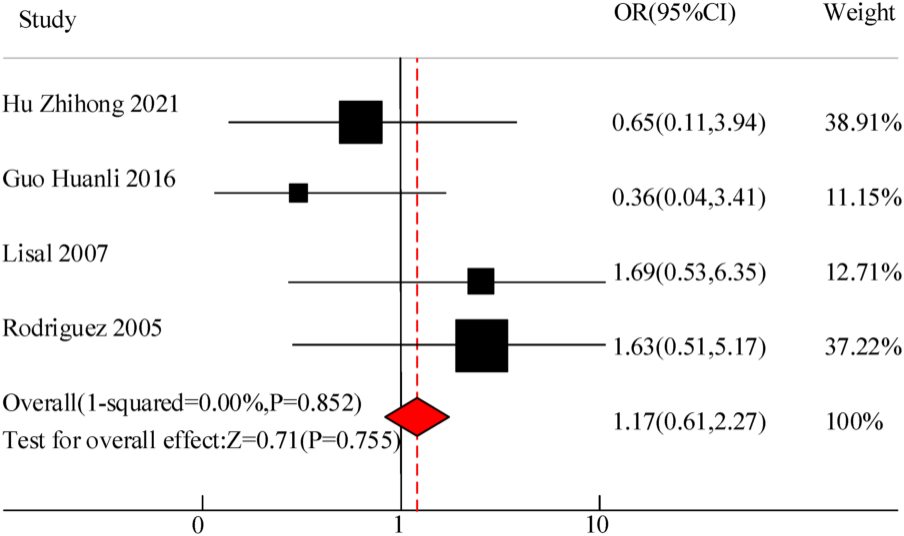
Adverse reaction forest Map.

This study included a total of 4 studies to compare the adverse reaction rates of VitA adjuvant treatment for pediatric pneumonia. The results showed homogeneity in the appearance of untowards effects between C and the experimental group (*I*^2^ = 0%, *P *= 0.852), so a fixed effects model was used for comprehensive evaluation. The conclusion shows that there is meaningless discrepancy in the occurrence of infaust effects between using VitA as an adjuvant therapy and C without VitA [OR = 1.17, 95% CI = (0.61, 2.27)], *P *> 0.05 (Figure 4).

#### 3.3.3 Hospital stay

**Figure 5:**
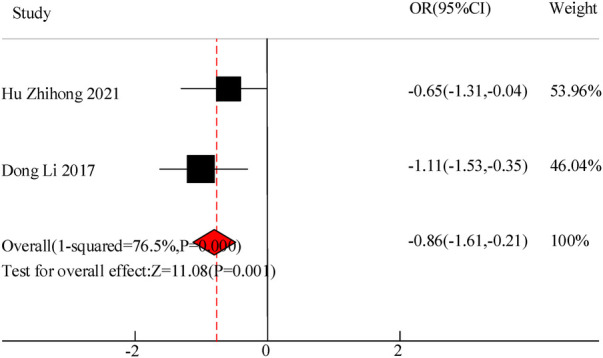
Forest map of hospitalization time.

Two studies were included in this study to compare the hospitalization time of VitA assisted treatment for pediatric pneumonia. The results showed heterogeneity in hospital stay between C and the experimental group (*I*^2^ = 76.5%, *P *= 0.000), so a random effects model was used for comprehensive evaluation. The conclusion shows that there is a significant difference in hospital stay between patients receiving VitA adjuvant therapy and those without VitA [MSD = −0.86, 95% CI = (–1.61, −0.21)], *P *< 0.05(Figure 5).

#### 3.3.4 The disappearance time of lung rale

**Figure 6:**
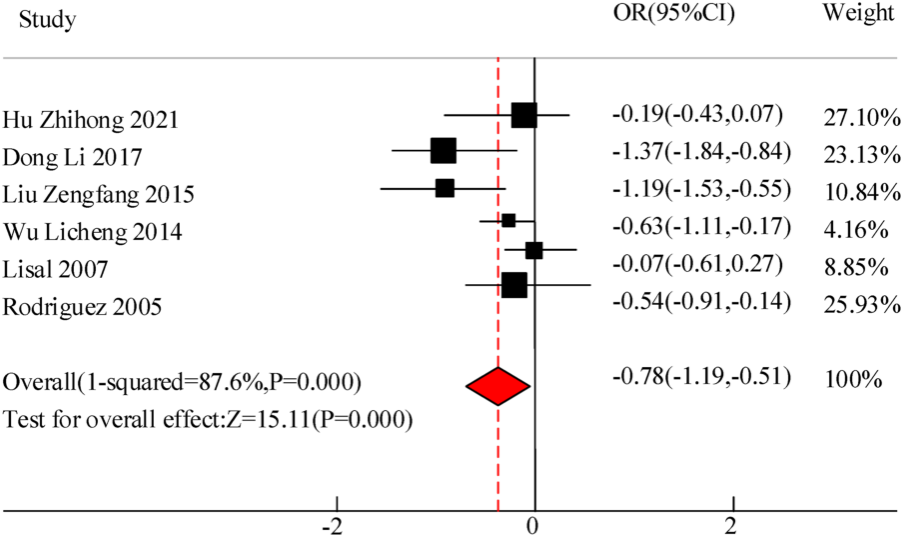
Forest map of lung rale disappearance time.

A total of 6 studies were included in this study to compare the disappearance time of lung rales in the adjuvant treatment of pediatric pneumonia with VitA. The results showed heterogeneity in the disappearance time of lung rales between the C and experimental groups (*I*^2^ = 87.66%, *P *= 0.000), so a random effects model was used for comprehensive evaluation. The conclusion shows that there is a significant difference in the disappearance time of lung rale between patients treated with VitA adjuvant therapy and those without VitA [MSD = −0.78, 95% CI = (−1.19, −0.51)], *P *< 0.05 (Figure 6).

#### 3.3.5 Cough duration

**Figure 7:**
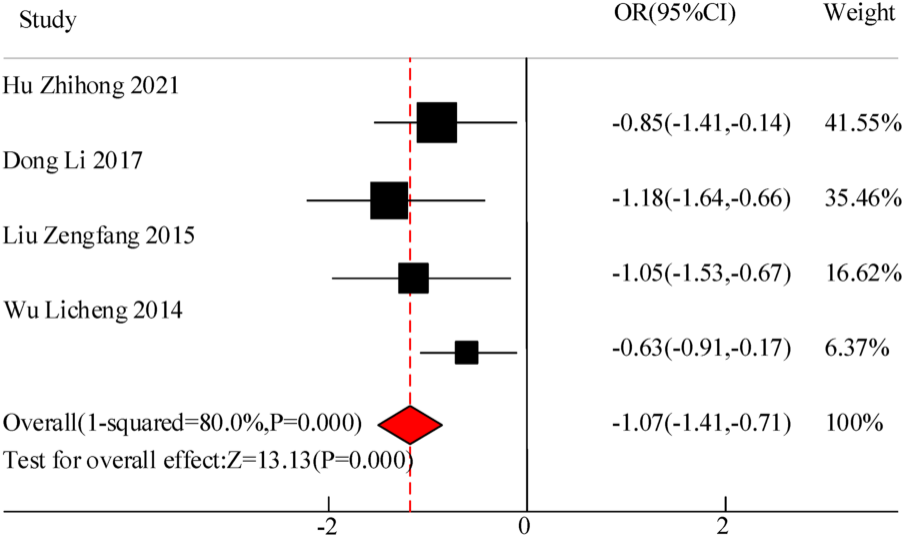
Forest map of cough duration.

This study included a total of 4 studies to compare the cough duration of VitA assisted treatment for pediatric pneumonia. The results showed heterogeneity in cough duration between the C and experimental groups (*I*^2^ = 80.0%, *P *= 0.000), so a random effects model was used for comprehensive evaluation. The conclusion shows that there is a significant difference in the duration of cough between patients treated with VitA adjuvant therapy and those without VitA [MSD = −1.07, 95% CI = (−1.41, −0.71)], *P *< 0.05 (Figure 7).

#### 3.3.6 Duration of fever

**Figure 8:**
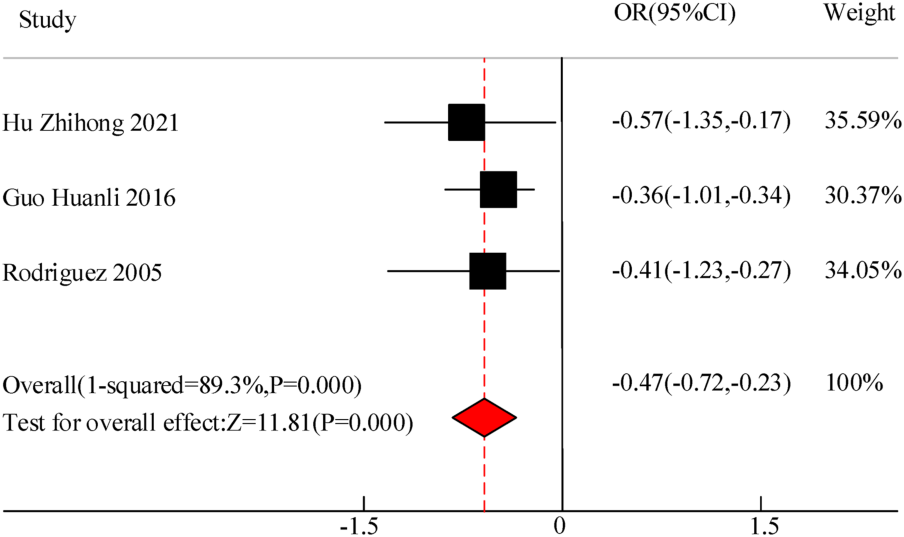
Forest map of fever duration.

Three studies were included in this study to compare the duration of fever in the adjuvant treatment of pediatric pneumonia with VitA. The results showed heterogeneity in the duration of fever between C and the experimental group (*I*^2^ = 89.3%, *P *= 0.000), so a random effects model was used for comprehensive evaluation. The conclusion shows that there is a difference in the duration of fever between C treated with VitA adjuvant therapy and C without VitA [MSD = −0.47, 95% CI = (−0.72, −0.23)], *P *< 0.05 (Figure 8).

#### 3.3.7 Inflammation levels (IL-6, IL-10)

**Figure 9:**
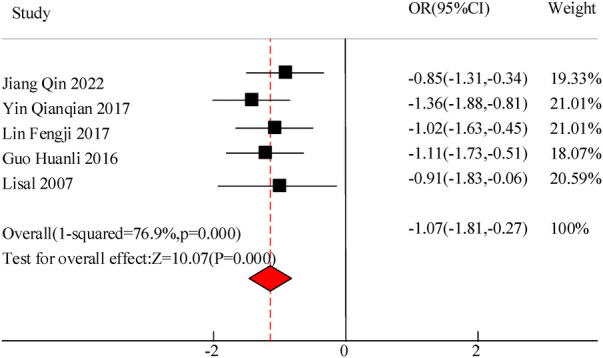
IL-6 level comparison forest map.

**Figure 10:**
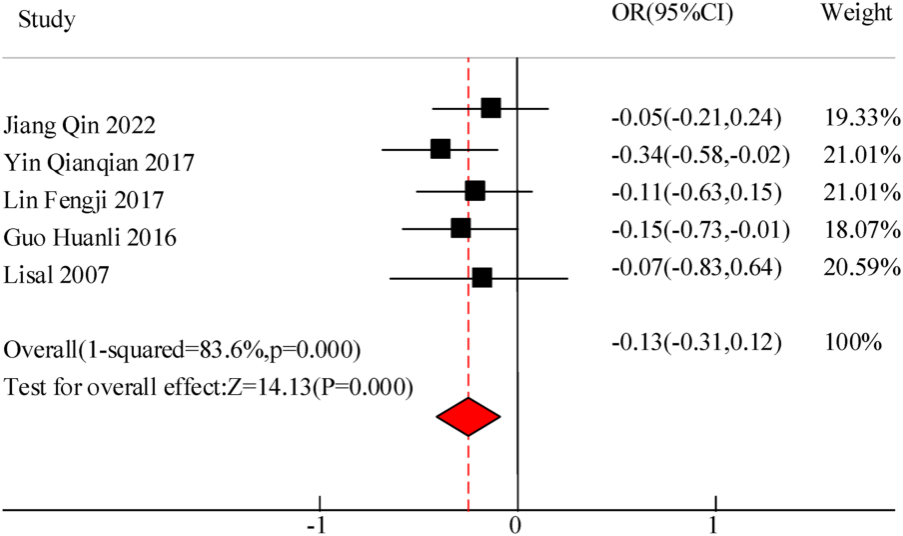
IL-10 level comparison forest Map.

This study included a total of 5 studies to compare the inflammatory levels of VitA adjuvant therapy for pediatric pneumonia. The results showed heterogeneity in IL-6 and IL-10 levels between the C and experimental groups (*I*^2^ = 76.9%, *P *= 0.000) and (*I*^2^ = 83.6%, *P *= 0.000), so a random effects model was used for comprehensive evaluation. The conclusion shows that there is a significant difference in IL-6 levels between C treated with VitA adjuvant therapy and C treated without VitA [MSD = −1.07, 95% CI = (−1.81, −0.27)], *P* < 0.05. There was a significant difference in IL-10 levels between C treated with VitA and C treated without VitA [MSD = −0.13, 95% CI = (−0.31, 0.12)], *P *< 0.05 (Figures 9, 10).

### 3.4 Sensitivity analysis

Sensitivity analysis was conducted on indicators such as clinical total response rate, adverse reactions, length of hospital stay, disappearance time of lung rale, duration of cough, duration of fever, IL-6 levels, and IL-10 levels. And to verify by comparing fixed and random models. The sensitivity analysis results show that the random effects and fixed effects of the six indicators all differ by less than 5%. This consistency indicates that the results of the included literature under different models are relatively stable and not affected by specific models. At the same time, the overlap range of the 95% confidence interval is large, which also supports this conclusion. Therefore, it can be considered that this study is relatively robust in terms of inclusion in the literature, and its results have high credibility and reliability (Table 3).

**Table 3:** Sensitivity analysis results.

Index	Random effect [SMD(95%CI)]	Fixed effect [OR(95%CI)]
Clinical total effective rate	3.11 (2.71, 4.41)	3.07 (2.81, 4.27)
Adverse reactions	1.13 (0.53, 2.51)	1.17 (0.61, 2.27)
Hospital stay	−0.86(−1.61, −0.21)	−0.91(−1.71, −0.13)
The disappearance time of lung rale	−0.78(−1.19, −0.51)	−0.80(−1.13, −0.48)
Cough duration	−1.07(−1.41, −0.71)	−1.03(−1.47, −0.73)
Duration of fever	−0.47(−0.72, −0.23)	−0.50(−0.70, −0.26)
IL-6 level	−1.07(−1.81, −0.27)	−1.04(−1.71, −0.31)
IL-10 level	−0.13(−0.31, 0.12)	−0.15(−0.35, 0.17)

## 4 Discussion

VitA is a lipid soluble vitamin that plays an important physiological function in the human body. VitA is an essential substance in processes such as visual adaptation, immune regulation, reproduction, and cell differentiation (13). VitA plays a particularly important role in the growth of children. In early childhood, VitA also occupies a vital position in bone growth and immune system development, helping infants and young children develop their immune function normally, improving their body's resistance, and preventing infections and diseases (14). In addition, the lung is an important target organ for VitA. VitA and its related metabolites are of great significance for the differentiation, proliferation, and development of lung tissue, as well as the repair of lung tissue damage (15). Although VitA plays many important physiological functions in the human body and plays an indispensable role in the growth and development of children and the immune system, its potential toxicity cannot be ignored. When the body ingests too much VitA, excessive VitA can reduce the stability of cell membranes and lysosomes, leading to the release of intracellular enzymes outside the cell and causing damage to surrounding tissues. In addition, excessive VitA can also cause functional damage to multiple organs. Excessive VitA may lead to dry skin, scaling, bone lesions, and brain damage. Pediatric MPP is a common respiratory infection caused by Mycoplasma bacteria, which mainly affects children. The symptoms of pediatric MPP include fever, cough, shortness of breath, nasal congestion, etc. In severe cases, it may lead to pneumonia and pulmonary complications. At present, studies have shown that VitA supplementation may help reduce the incidence rate and severity of pediatric MPP (16). The immunomodulatory effect of VitA may help enhance children's immune function, enhance their resistance to pathogens such as mycoplasma, and thus reduce the risk of infection (17). In addition, the maintenance of the mucosal barrier by VitA also helps to prevent pathogens from invading the respiratory tract and reduce the occurrence of respiratory infections.

In the 10 articles included in the meta-analysis of this study, VitA adjuvant therapy was used. The control group received conventional treatment mainly including cough and asthma suppressants for the children. The specific treatment methods included intravenous infusion of azithromycin, intravenous infusion of ambroxol, and other conventional pediatric pneumonia drugs. There were certain differences in medication with relevant literature, but the effect of VitA adjuvant therapy was not significant. The results of this meta-analysis indicated that there was a significant difference in the overall clinical efficacy of using VitA as an adjuvant therapy compared to C without VitA [OR = 3.07, 95% CI = (2.81, 4.27)], *P *< 0.05. However, there was no significant difference in the adverse reaction rate between the use of VitA as an adjuvant therapy and C without VitA [OR = 1.17, 95%CI = (0.61, 2.27)], *P *> 0.05. The analysis results of this article are consistent with the research results of Hu Zhihong and Guo Huanli. Hu Zhihong's research on 300 cases of pediatric MPP mentioned that the total effective rate of O's VitA adjuvant therapy reached 94.67%, much higher than C80.67% (*P *= 0.000 < 0.05); The incidence of adverse reactions was 2.67%, which was not significantly different from C's 4.67% (*P *= 0.520 > 0.05). This indicates that VitA adjuvant therapy can significantly improve the effective rate of pediatric MPP without increasing the occurrence of adverse reactions. Clinical application of VitA has high safety. This meta-analysis validated that the hospitalization time [MSD = −0.86, 95% CI = (−1.61, −0.21)], lung rale disappearance time [MSD = −0.78, 95% CI = (−1.19, −0.51)], cough duration [MSD = −1.07, 95% CI = (−1.41, −0.71)], and fever duration [MSD = −0.47, 95% CI = (−0.72, −0.23)] were significantly lower than those of C. Consistent with the RCT research results of Hu Zhihong and Dong Li, intravenous infusion of azithromycin and ambroxol in pediatric patients, as well as the use of VitA as an adjunct therapy, has significant advantages in treating pediatric MPP. The reasons may involve the following aspects: VitA plays an essential regulatory role in the immune system, especially in mucosal immunity. Mycoplasma pneumonia is a respiratory infection disease, and the mucosa serves as a protective barrier for the respiratory tract (18). VitA helps to maintain the integrity of the mucosal barrier, enhance the immune function of mucosal cells, enhance resistance, and thereby reduce the risk of pathogens invading the respiratory tract, reducing the degree and duration of infection (19). VitA has a certain anti-inflammatory effect, which can regulate inflammatory reactions, inhibit the production of inflammatory factors and the transmission of cellular signals. In mycoplasma pneumonia, the invasion of pathogens can trigger an inflammatory response in the respiratory tract, leading to worsening and persistence of symptoms (20). The anti-inflammatory effect of VitA can alleviate respiratory inflammation, alleviate symptoms, and thereby reduce the duration of lung rales, cough, and fever. VitA is also an important regulatory factor for cell differentiation and proliferation. During mycoplasma infection, VitA may promote repair and recovery by regulating the proliferation and differentiation of damaged lung tissue cells, which helps to accelerate the disappearance of lung rales and alleviate coughing and fever (21). Although the role of VitA in the immune system and respiratory infections has been preliminarily confirmed, the specific treatment effect still needs to consider the impact of individual differences on the efficacy of VitA. Moreover, whether long-term use of VitA will lead to other health problems, such as toxic reactions caused by excessive VitA, is also an issue that future research needs to pay attention to.

The results of this meta-analysis also indicate that the use of VitA adjuvant therapy can significantly reduce IL-6 [MSD = −1.07, 95% CI = (−1.81, −0.27)] and IL-10 [MSD = −0.13,95% CI = (−0.31, 0.12)] levels. This indicates that the application of VitA in pediatric MPP also has the effect of reducing inflammatory response. This may be because IL-6 and IL-10 are both inflammatory factors that play a role in regulating inflammatory responses in the immune system; In pediatric MPP, the invasion of pathogens can lead to the activation of the immune system, releasing a large number of inflammatory factors, and subsequently triggering an inflammatory response; The application of VitA may inhibit the production of IL-6 and IL-10, thereby alleviating inflammatory reactions and reducing the degree of inflammation. In addition, VitA has a certain regulatory effect on the function of immune cells (ImCs). Research has shown that VitA can promote the activity of certain ImCs, like macrophages and dendritic cells, while inhibiting the activity of other ImCs, e.g., T and B lymphocytes (22). This regulatory effect may help balance the inflammatory response, reduce excessive immune response, and thus reduce releasing the inflammatory factors. VitA may also cause the occurrence and progression of inflammatory reactions by regulating the activity of cellular signaling pathways. It can affect multiple signaling pathways, including NF-κB, MAPK, JAK-STAT, etc., to affect the production of inflammatory factors and the activity of ImCs, thereby regulating the intensity and duration of inflammatory reactions. In actual clinical environments, as the condition progresses and treatment progresses, the levels of inflammatory factors may go through a process of increasing to gradually decreasing. This means that simply comparing the average levels of inflammatory factors before and after treatment may not fully reflect the true role of VitA in regulating inflammatory response. Therefore, to more accurately evaluate the impact of VitA on inflammatory factors, future research needs to pay more attention to the dynamic process of changes in inflammatory factor levels over time.

Based on the above research results, this meta-analysis clarifies the clinical efficacy of vitamin A as an adjuvant therapy for pediatric MPP and its impact on inflammation levels. Through the analysis of 10 RCT studies included, it was found that the utilization of vitamin A could significantly rise the clinical symptoms of pediatric MPP cases, shorten hospitalization time, promote the disappearance of lung rales, and alleviate cough and fever symptoms. At the same time, vitamin A adjuvant therapy also showed a significant reduction in IL-6 and IL-10 levels, demonstrating its regulatory effect on inflammatory response. However, this study also has some limitations, including a small number of included studies, small design and sample size in some studies, and possible unknown bias factors. Therefore, in clinical practice, it is recommended to carefully evaluate the efficacy and safety of vitamin A adjuvant therapy, and comprehensively consider the individual characteristics of the patient, following the doctor's advice and medication guidance. In order to further validate the efficacy and mechanism of action of vitamin A in pediatric MPP, more high-quality, large-scale clinical studies are needed to confirm it. In addition, further exploration is needed on the optimal dosage and timing of vitamin A administration. It is expected that the research findings can give a certain reference basis for the treatment of pediatric MPP, and provide beneficial insights for clinical practice and further research.

## Data Availability

The data that support the findings of this study are available on request from the corresponding author.
